# Effects of biomass reducing agent on magnetic properties and phase transformation of Baotou low-grade limonite during magnetizing-roasting

**DOI:** 10.1371/journal.pone.0186274

**Published:** 2017-10-17

**Authors:** Kai Zhang, Xiu li Chen, Wen chao Guo, Hui juan Luo, Zhi jun Gong, Bao wei Li, Wen fei Wu

**Affiliations:** 1 School of Energy & Environment, Inner Mongolia University of Science & Technology, Baotou, Inner Mongolia Autonomous Region, China; 2 Key Laboratory of Integrated Exploration of Bayun Obo Multi-Metal Resources, Baotou, Inner Mongolia Autonomous Region, China; 3 Faculty of Biological Science and Technology, Baotou Teachers College, Baotou, Inner Mongolia Autonomous Region, China; CAS, CHINA

## Abstract

Biomass was used as reducing agent to roast the Baotou low-grade limonite in a high temperature vacuum atmosphere furnace. The effect of calcination temperature, time and ratio of reducing agent on the magnetic properties of calcined ore was studied by VSM. The phase and microstructure changes of limonite before and after calcination were analyzed by XRD and SEM. The results show that in the roasting process the phase transition process of the ferrous material in limonite is first dehydrated at high temperature to formα-Fe_2_O_3_, and then it is converted into Fe_3_O_4_ by the reduction of biomass. With the increase of calcination temperature, the magnetic properties of the calcined ore first increase and then decrease. When the temperature is higher than 650°C, Fe_3_O_4_ will become Fe_2_SiO_4_, resulting in reduced the magnetic material in calcined ore and the magnetic weakened. The best magnetization effect was obtained when the roasting temperature is 550°C, the percentage of biomass was 15% and the roasting time was 30min. The saturation magnetization can reach 60.13emu·g^-1^, the recovery of iron was 72% and the grade of iron was 58%.

## 1. Introduction

Steel is the most widely used metallic material in human life. It has been extensively used in various fields and is an indispensable strategic material. With the development of the global economy and the constant consumption of resources, the high-grade iron ore which is easy to beneficiation is facing a severe shortage. The abundant low-grade limonite becomes one of the important raw material sources to produce iron and steel. The limonite is an argillaceous substance containing a series of iron hydroxides, the majority of them exist in the form of the goethite series, and containing a large amount of crystalline water. Because of its loose structure, easy to slime, small specific gravity and poor flotation performance, the use of hydrometallurgy or pyrometallurgy technology to obtain the high-grade ore is complicated, high energy-consumption and heavy-pollution[1–3].The limonite in Guyang, Baotou, Inner Mongolia Autonomous Region in China, is large in reserves, low in grade, complicated in distribution and uneven in size. The limonite in this area mainly in the form of goethite, water goethite and iron oxides containing different content of crystalline water, as well as a mixture of argillaceous substances. The symbiotic impurity minerals are mainly silicate. The common method of treating refractory iron ore with complex structure is magnetic roasting[4–8] which can make weak magnetic iron oxide convert into strong magnetic iron oxide[9–11], and then iron-bearing substances is separated from impurities by magnetic separation. The method of magnetic roasting in the industry for iron ore is mainly the reduction roasting of gas or coal as reducing agent[12]. However, due to the limitation of the resources and technology, the roasting process of coal as reducing agent is commonly used at home and abroad. Under the outstanding situation of environmental problems and the energy problems in the world, the problem of the high energy consumption and heavy pollution during the roasting process of coal as reducing agent should not be ignored. How to make the production of steel be efficient, low carbon and green is concerned by many researchers. The use of clean energy instead of non-renewable energy coal as a reducing agent is of great significance on magnetic roasting of iron ore. The feasibility of biomass as a non-toxic and renewable carbon neutral solid fuel replacing fossil fuels has been explored by many researchers. W.U. Yan et al[13] studied the reduction roasting of goethite at 550~600°C for 1h with the biomass as reducing agent found that the magnetic properties of minerals were effectively strengthened. Y.B. Wang et al[14] studied the reductive magnetic roasting of limonite. It was found that using biomass as reducing agent could prevent the reduction process from bonding, the effect of magnetization was better than the lignite as reducing agent, and the roasting temperature was lower by more than 100°C.

The roasting process of iron ore has been well known to many researchers. But phase changes of mineral phases are diverse in the roasting process because of the different composition and structure of limonite. At present, research on the mechanism of the phase transformation of the limonite roasted with biomass as reducing agent, coming from Guyang, Baotou, Inner Mongolia Autonomous Region in China, is rare. Therefore, the purpose of this study using biomass as reducing agent to reduce limonite is not only to investigate the influence of different factors on magnetic characteristics of minerals and the change regulation of iron phase in roasting process, but also to contribute to improvement of the recovery rate of iron.

## 2. Materials and methods

### 2.1 Materials

**Fig 1:**
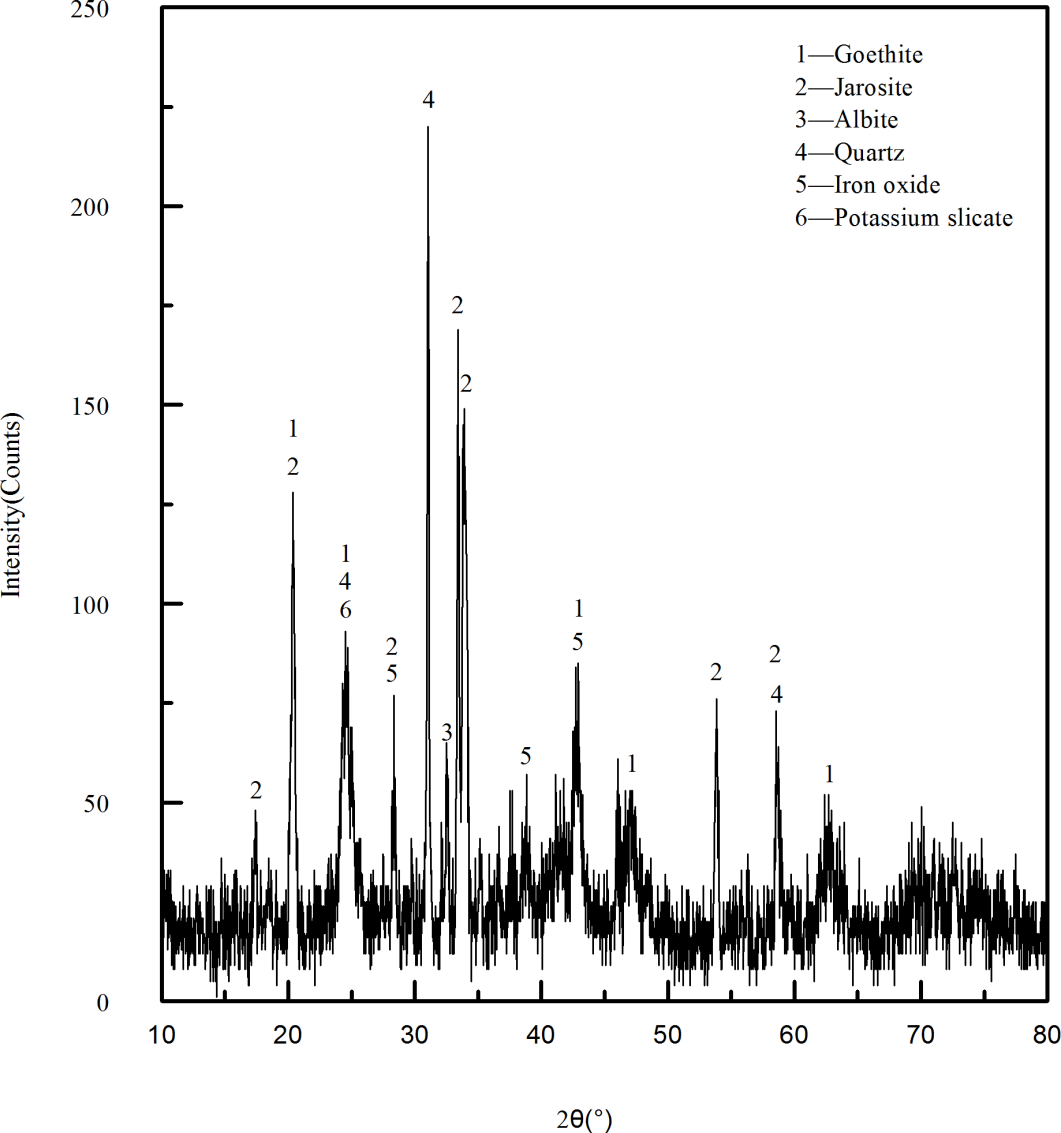
The XRD pattern of raw ore.

**Fig 2:**
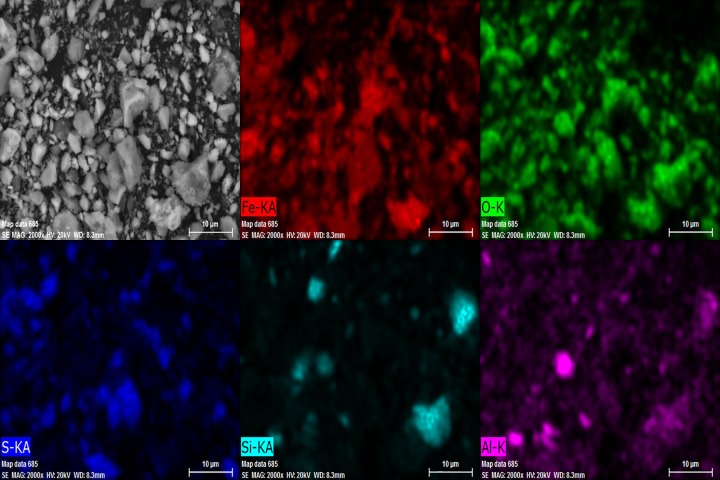
SEM image of raw ore, and elements distribution in the limonite ore sample.

Limonite ore sample was collected from Baotou City, Inner Mongolia Autonomous Region, China. The sample ground to a particle size of less than 0.178mm was studied by X-Ray diffraction(XRD) and scanning electron microscopy(SEM). The chemical composition analysis of raw ore is shown in Table 1. It can be seen from Table 1 that the sample belongs to low-grade limonite, and the content of other gangue(mainly SiO_2_) is 32.69%,it also contains a small amount of alkali metal compounds. The XRD pattern of the raw ore in Fig 1 indicated that the mineral phase of iron-bearing materials in raw ore were mainly goethite(Fe(OH)) and jarosite (KFe_3_(SO_4_)_2_(OH)_6_·xH2O), and the phase of main gangue is quartz. Fig 2 presents the scanning electron microscopy(SEM) image and the distribution map of major elements in the region. The bright part of color in the distribution map is the main area of the corresponding element, it can be seen from the picture that iron-bearing materials is evenly distributed and their size is relatively uniform in the raw ore. In addition there were some substances of different sizes containing silicon and aluminum wrapped by other minerals, and the distribution of substances containing silicon is relatively concentrated and have clear boundaries with other minerals. The biomass used in the experiment was taken from the local agricultural area, and the industrial analysis of biomass was shown in Table 2.

**Table 1:** Chemical phase analysis (%).

TFe	FeO	SiO_2_	Na_2_O	MgO	Al_2_O_3_	P	S	F	K_2_O	CaO
33.58	1.94	32.69	1.57	0.34	3.65	0.13	2.15	0.06	1.69	0.72

**Table 2:** The industrial analysis of biomass (%).

moisture	Ash	volatile matter	fixed carbon
8.93	2.52	75.14	13.41

### 2.2 Methods

The area where the sample used in this test is collected are not owned by the private owner, since all the land in China is owned by the Chinese state, and we study the characteristics of the region's ore after the consent of the Chinese Inner Mongolia Autonomous Region government. In addition, the field studies did not involve endangered or protected species.

The limonite was crushed and ground to a particle size less of than 0.178mm by the ball grinder after drying 5h in a constant-temperature dry box at 105°C. Then a certain amount of limonite powder was thoroughly mixed with biomass particle milled to a particle size of less than 0.178mm in the crucible, and the crucible was placed into the high temperature vacuum atmosphere furnace. The samples were kept inside the furnace after setting desired temperature and residence time, and the heating rate is set to 20°Cmin^-1^ in all experiments. On completion of the experiment, the sample was taken out when the temperature was cooled to room temperature. The calcined ore was ground to a particle size of less than 0.178mm and was packed into a marked bag for further analysis. The iron-bearing substance was separated from other impurity by magnetic separation after analyzing the characteristics and the recovery of iron is calculated. The effects of different roasting temperature, the content of biomass and the roasting time on limonite during magnetization roasting process were studied. When the one parameters were measured, the other parameters were unified.

### 2.3 The characterization techniques for mineral analysis

The magnetism of the ore was studied by the hysteresis loop curve obtained at room temperature through a vibrating sample magnetometer(Model: Lake Shore 7410 VSM) under a maximum magnetic field strength of about 3T.The mineral phase of ore was studied by X-ray Diffractometer(Model: Rigaku-TTR Ⅲ) using Cu Kα radiation at a scanning rate of 4°·min^-1^ from 10 to 80°.Themorphology and microstructure of the ore were investigated by scanning electron microscopy(Model: TescanMIRA3/Oxford X-Max20).The Thermo-gravimetric(TG) and Differential Scanning Calorimetry(DSC) studies were carried out using the NetzschSta 449C equipment.

## 3. Results and discussions

### 3.1 The magnetism of the iron ore

**Fig 3:**
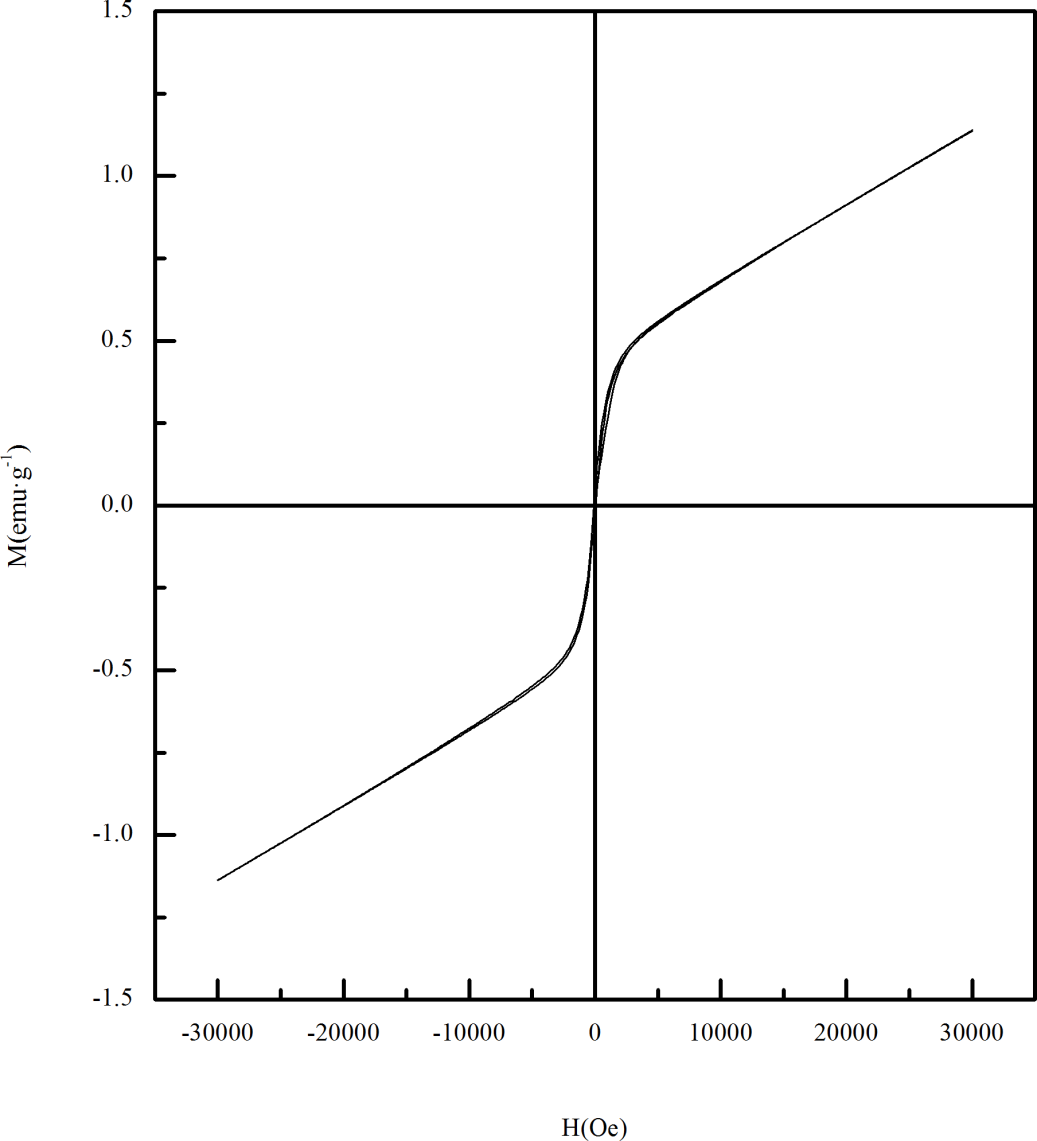
Hysteresis loop of raw ore.

**Fig 4:**
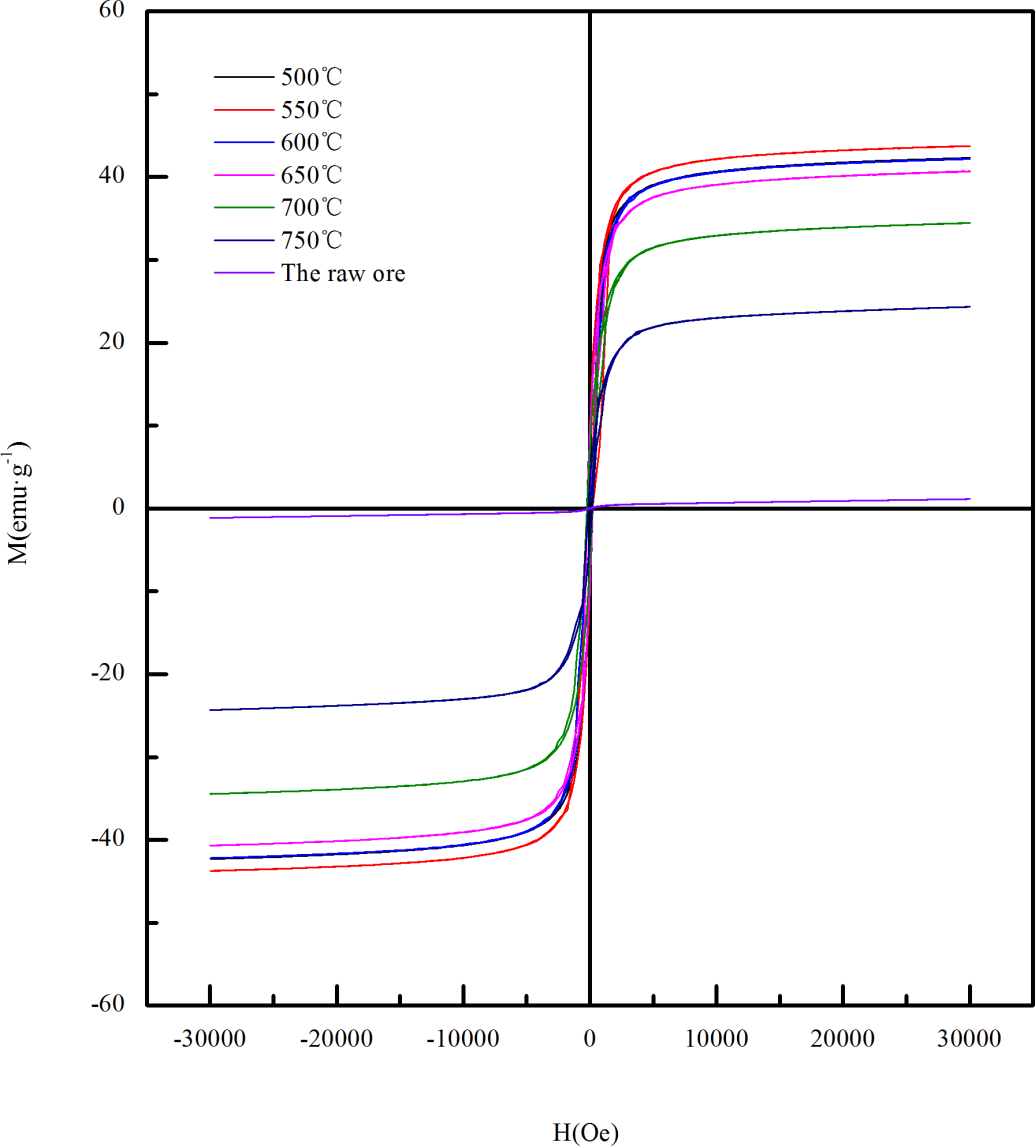
Hysteresis loop of calcined ore obtained under different temperatures.

In order to study the effect of magnetic roasting on limonite coming from Guyang, the VSM was used to detect the magnetic properties of the raw ore and the calcined ore obtained from different roasting conditions. Fig 3 shows the hysteresis loop curve of the raw ore obtained at room temperature. As can be seen from the picture that the magnetism of raw ore is weak, so it is difficult to achieve the desired recovery effect if the grinding-screening-magnetic separation is used directly. The hysteresis loop of calcined ore is shown in Fig 4. Compared with the hysteresis loop curve of raw ore, the hysteresis loop curve of calcined ore is consistent with the hysteresis loop curve of the typical strong magnetic material. This is because that iron-bearing materials in the limonite become α-Fe_2_O_3_ after dehydration at the high temperature, and then α-Fe_2_O_3_ was reduced to strong magnetic iron oxide. It can be seen from Fig 4 that there is a sharp increase in the magnetization at the beginning of magnetization stage with the increase of the applied magnetic field strength (H), and then a more stable magnetization (saturation magnetization Ms) is reached. But when the field strength of the external magnetic field is weakened, the magnetization of the sample decrease with the curve which is different from the starting magnetization curve. When H = 0T, the magnetization of the sample is not equal to 0. The residual magnetization of the sample is called remanence(Mr). When the magnetization of the sample is 0T, the intensity of the external magnetic field is called coercivity(Hc). The comparison shows that biomass reduction magnetization roasting can effectively change the magnetic properties of Guyang limonite ore, which is beneficial to subsequent magnetic separation.

#### 3.1.1 Influence of temperature on magnetic properties of calcined ore

**Fig 5:**
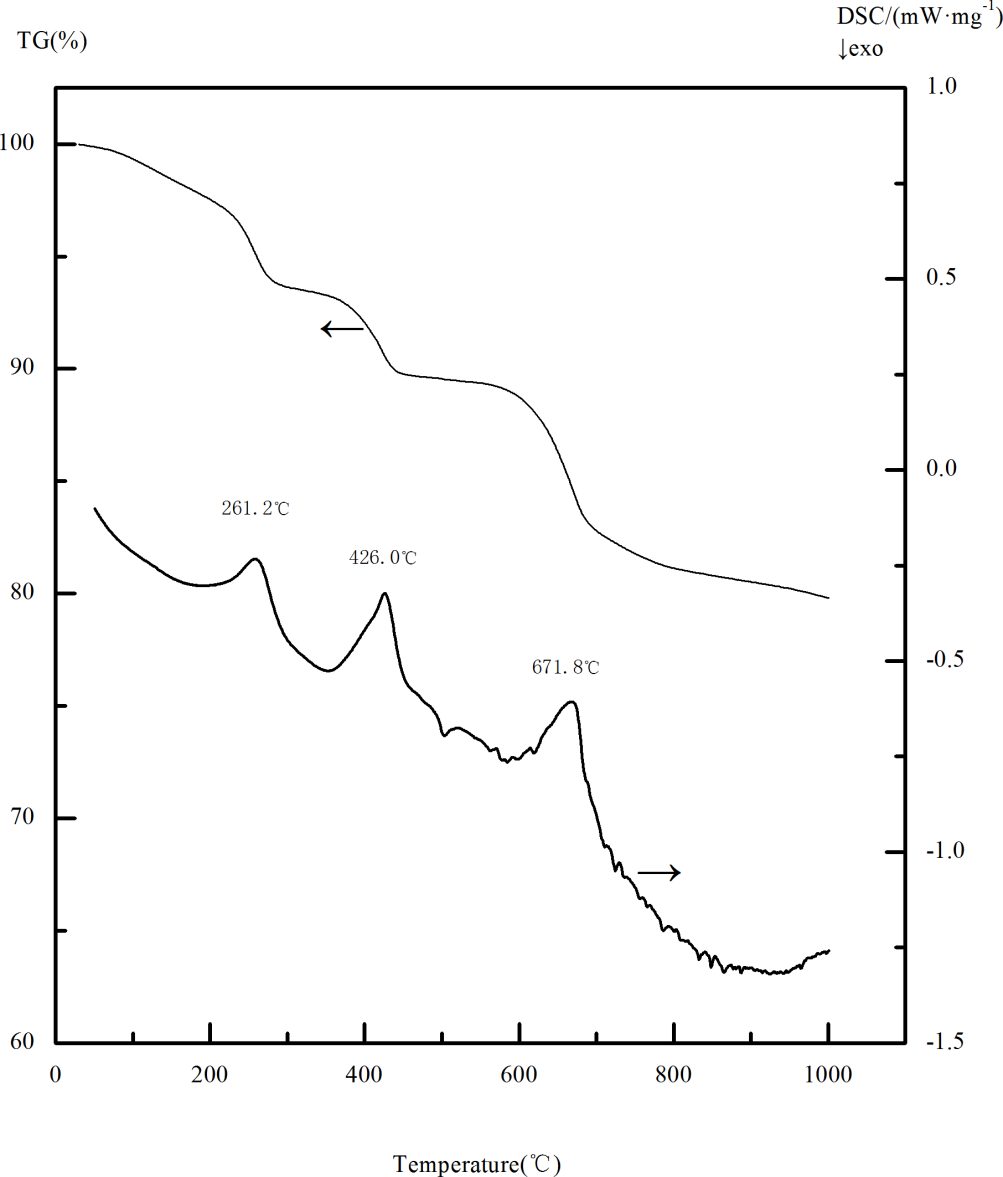
TG/DSC curve of the raw ore.

**Fig 6:**
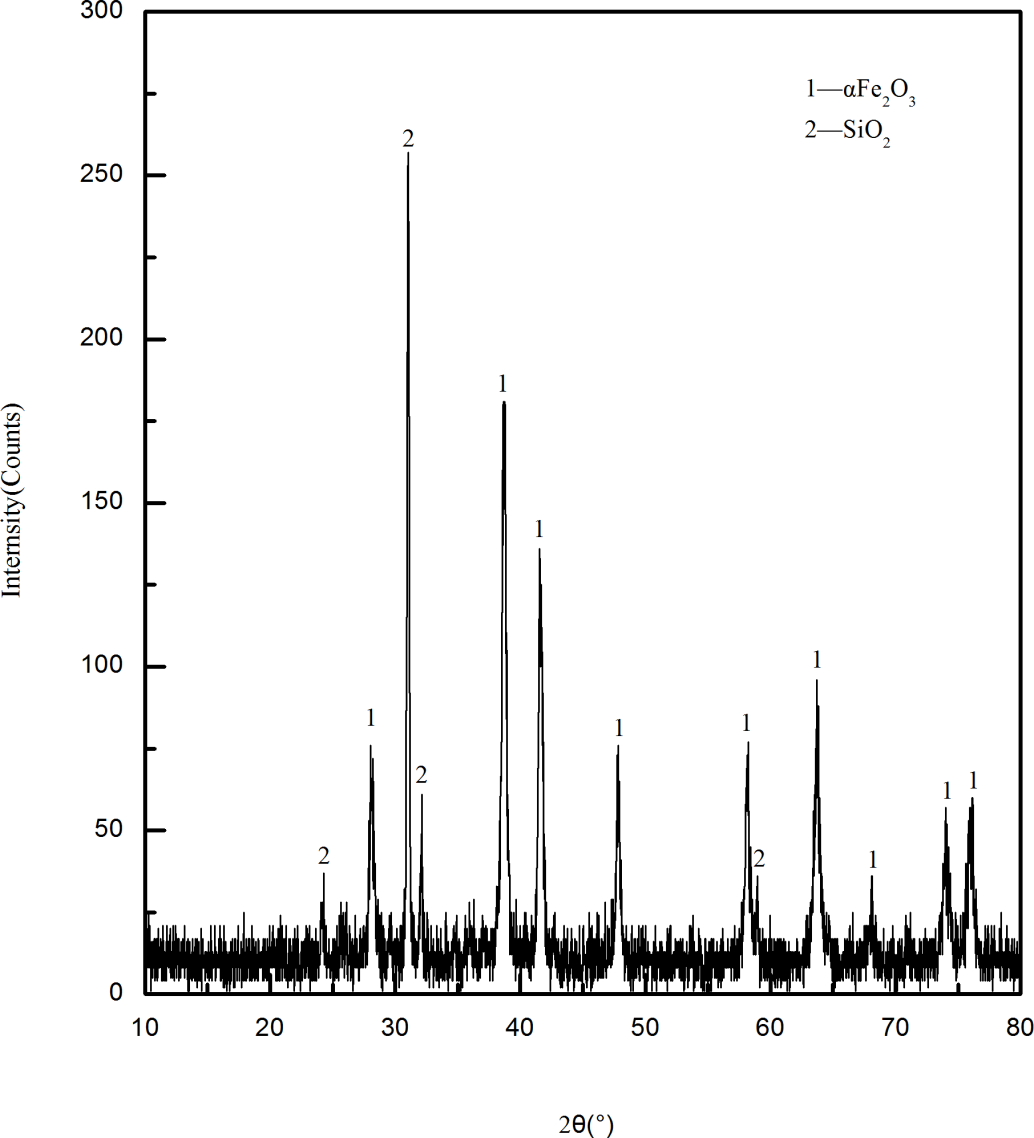
The XRD pattern of calcined ore obtained at 671.2°C.

**Fig 7:**
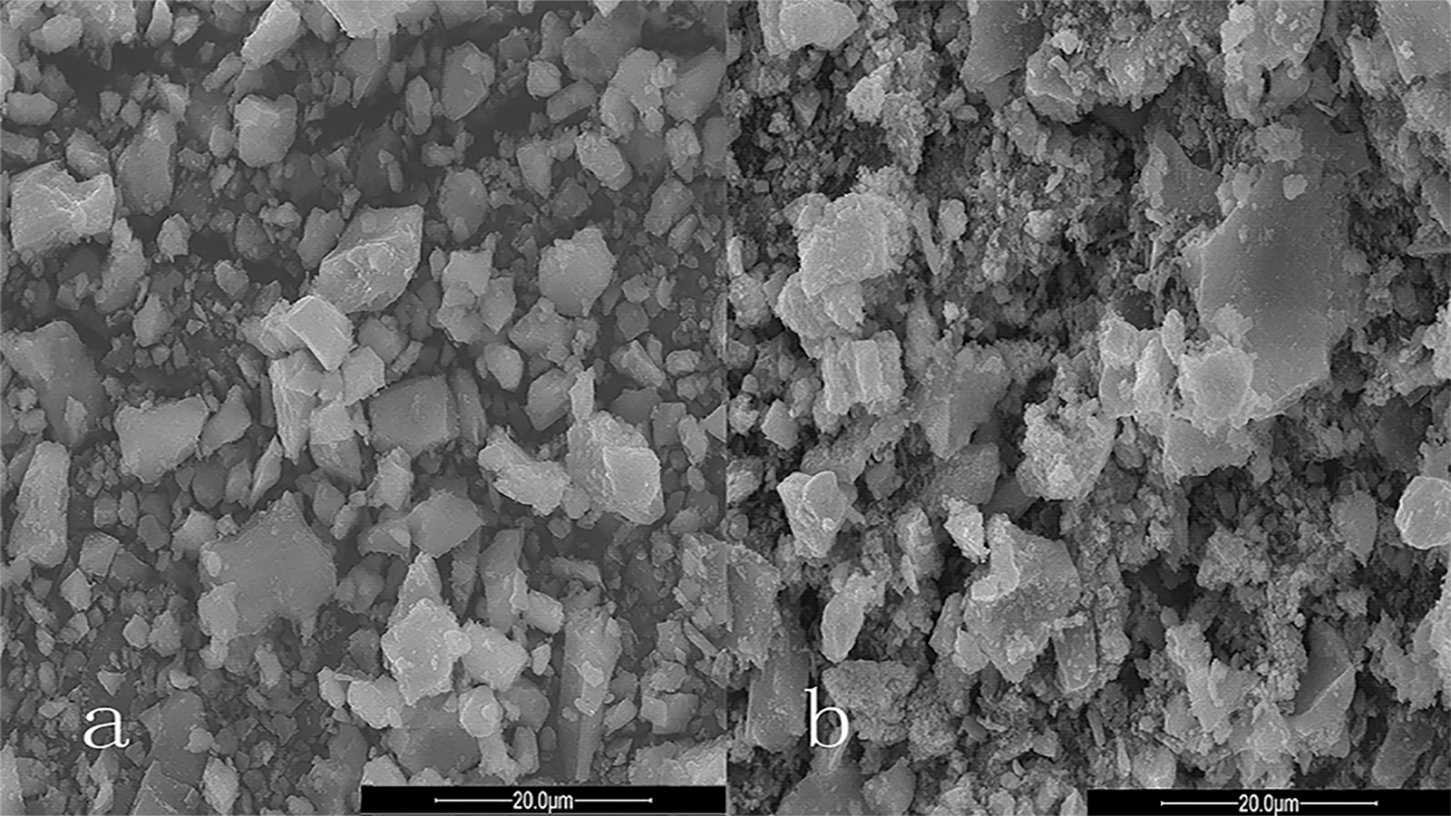
The SEM diagram (a:the raw ore, b:the calcined ore obtained at 671.2°C).

The temperature plays an important influence on the phase change of limonite during magnetic roasting. In order to determine the range of roasting temperature, thermo-gravimetric analysis of the raw ore was carried out at the heating rate of 20°Cmin^-1^. The TG/DSC curve was shown in Fig 5. As can be seen from the TG curve in Fig 5, there are three major weight losses in the heating process of limonite between 0~1000°C.The first weight loss started at 30°C and ended at 288.8°Cwith a total weight loss ratio was of 6.57%. The second weight loss started at 288.8°C and ended at 446.3°C. The total weight loss ratio was about 3.90%. And the third weight loss started at 446.3°C, and ended at 875.8°C. The total weight loss ratio was about 8.93%. In the DSC curve three endothermic peaks can be seen, it can be inferred that the endothermic peak at 261.2°C is due to the desorption of adsorbed water in mineral, and the endothermic peak at 426°C is owing to the removal of crystalline water from the minerals, as well as the endothermic peaks at 671.8°C is because the decomposition of jarosite into Fe_2_O_3_ and SO_2_ after losing the crystalline water. The above phenomena are similar to the results of P.C. Beuria et al[15] and W. Pan et al[16] on thermal decomposition characteristics of limonite and X.E. Cao et al[17] on the thermal decomposition of ammonium compounds. Fig 6 is the XRD diagram of the calcined ore obtained at 671.2°C. It can be seen that the main iron content in the mineral is α-Fe_2_O_3_, and the main impurity minerals are SiO_2_. It can be seen from the SEM of the mineral (Fig 7) that limonite produces a large amount of pores in the heating process and the structure becomes loose compared to the raw ore, which facilitates the diffusion of the reducing gas into the interior of the particles, enabling the limonite to be completely reduced to magnetite. Hence, the roasting temperature was set at 500~750°C by a comprehensive analysis. The iron mineral phase occur as follows during the process of decomposition[17, 18]:

<280°C:

$${KFe}_{3}{\left({SO}_{4}\right)}_{2}{\left(OH\right)}_{6}\cdot xH_{2}O\to {KFe}_{3}{\left({SO}_{4}\right)}_{2}{\left(OH\right)}_{6}+xH_{2}O$$ (1)

300~450°C:

$$2{KFe}_{3}{\left({SO}_{4}\right)}_{2}{\left(OH\right)}_{6}\to K_{2}SO_{4}+{Fe}_{2}{\left({SO}_{4}\right)}_{3}+2\alpha -{Fe}_{2}O_{3}+6H_{2}O$$ (2)

$$2FeO(OH)\to \alpha -{Fe}_{2}O_{3}+H_{2}O$$ (3)

600~750°C:

$$2{Fe}_{2}{\left({SO}_{4}\right)}_{3}\to 2\alpha -{Fe}_{2}O_{3}+6{SO}_{2}↑+3O_{2}↑$$ (4)

**Fig 8:**
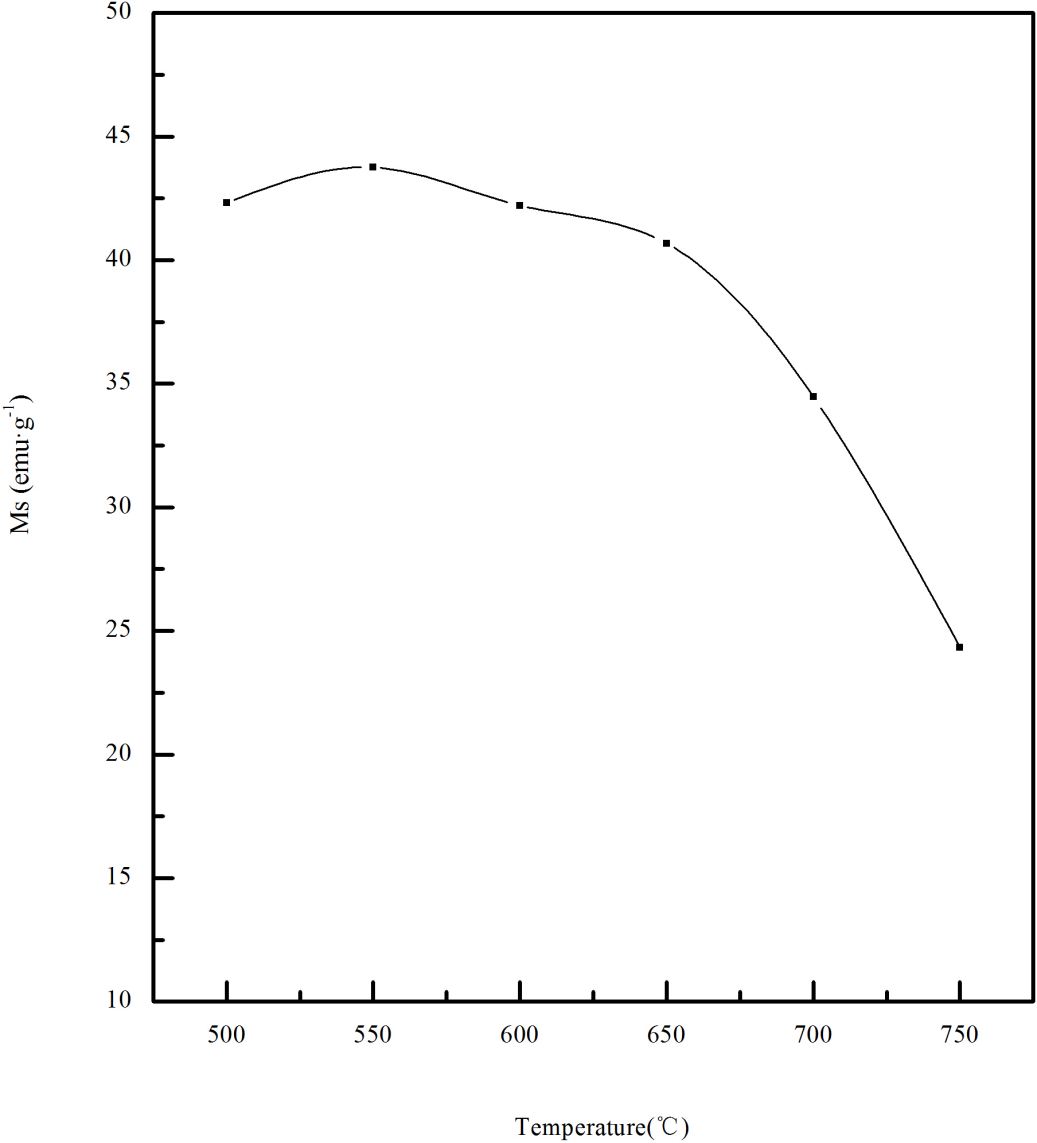
Effect of roasting temperature on saturation magnetization.

Fig 8 shows the relationships of the saturation magnetization of the calcined ore and the temperature between 500~750°C, the other conditions are that the content of the biomass to limonite is 25% and the roasting time is 45min. Compared with the magnetism of the raw ore, the reduction magnetization roasting of limonite using biomass as reducing agent can make the magnetism of minerals be obviously strengthened. The magnetism of the calcined ore was enhanced with the increase of temperature between 500~550°C.The maximum reached 43.75emu·g^-1^ at 550°C.When the temperature continued to rise, the magnetism of the calcined ore began to decrease. This may be because that with the increasing temperature, more Fe^2+^ has been produced to reduce the magnetic materials in the minerals, which weakens the magnetism of the minerals. The roasting effect of limonite is better at a temperature of 550°C.

#### 3.1.2 Influence of biomass content on magnetic properties of calcined ore

**Fig 9:**
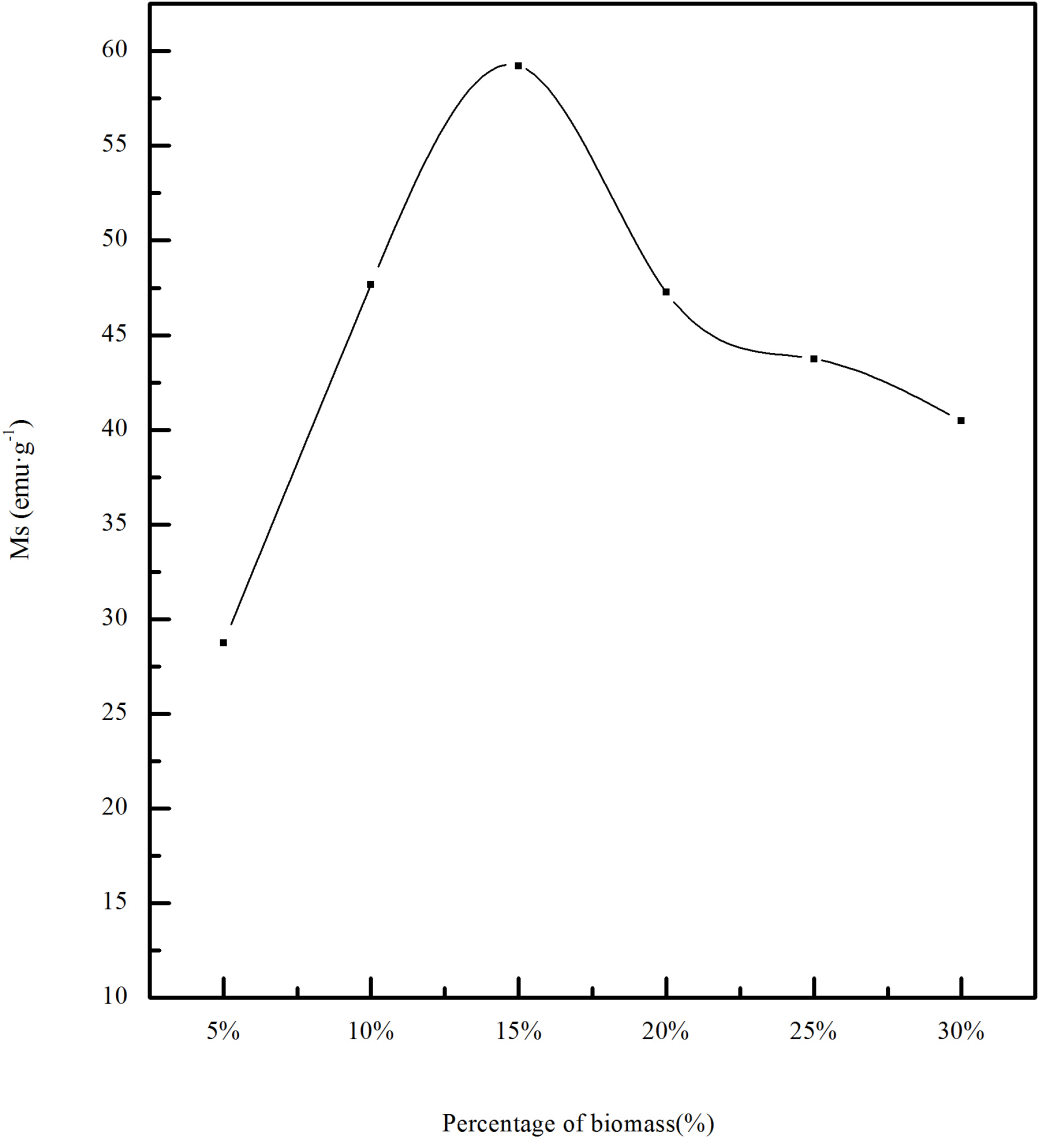
Effect of the content of biomass on saturation magnetization.

The relation between saturation magnetization and biomass content is shown in Fig 9. The roasting temperature is 550°C and the roasting time is 45min.It can be seen from the diagram that the magnetic properties of calcined ore first increase and then decrease with the increase of biomass content. Between 5%~15%, the magnetic properties of calcined ore rapidly increase. When the ratio of biomass is 15%, the magnetization is 59.23emu·g^-1^. When the content of the biomass continues to increase, the magnetic properties of the calcined ore gradually decrease. Therefore, the content of 15% is better for roasting of limonite.

#### 3.1.3 Effect of the roasting time on magnetic properties of calcined ore

**Fig 10:**
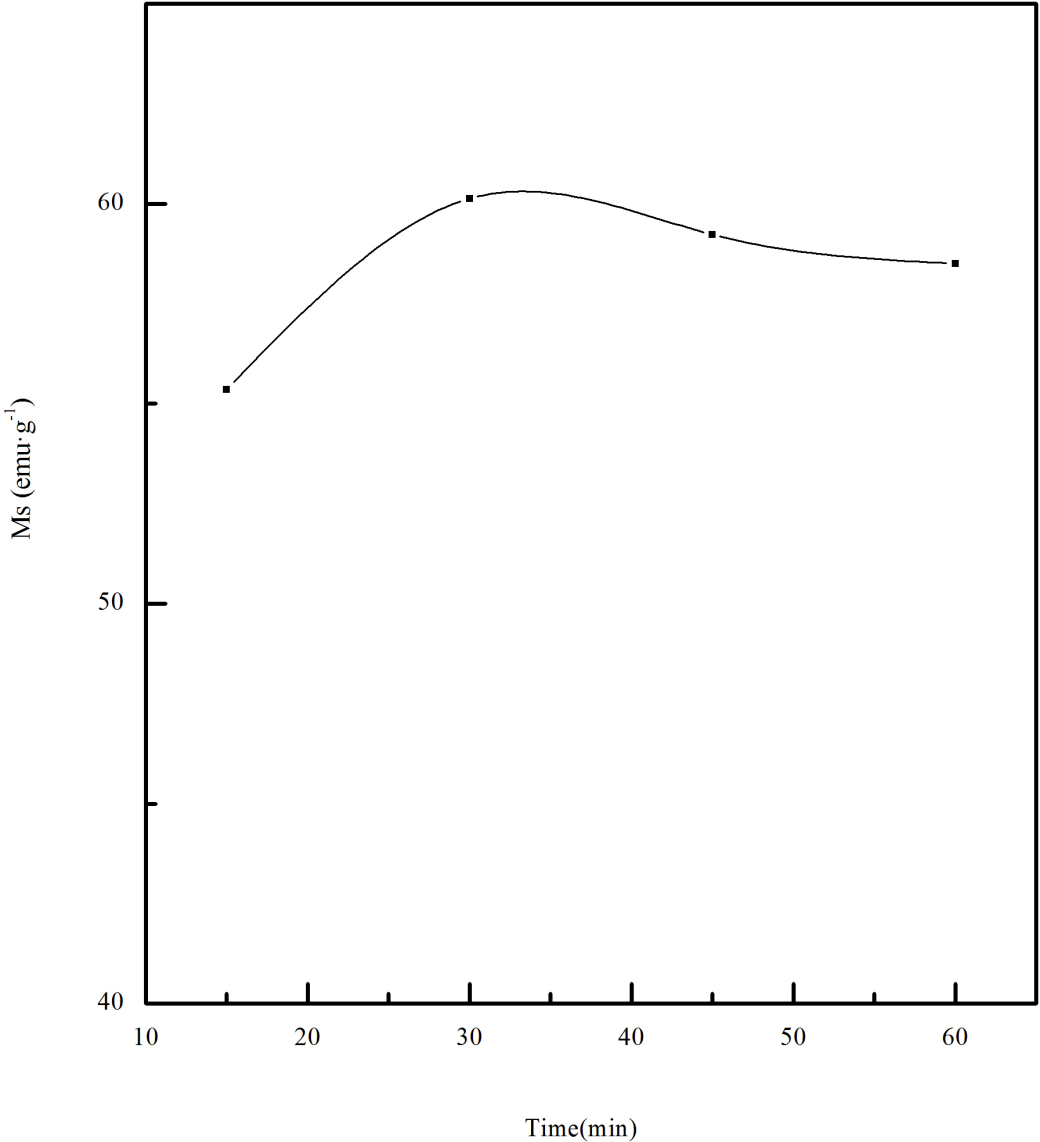
Effect of roasting time on saturation magnetization.

The effect of roasting time on the magnetic properties of calcined ore was investigated at a temperature of 550°C and the ratio of biomass is 15%. The relation between roasting time and saturation magnetization is shown in Fig 10. When the roasting time was extended from 15min to 30min, the magnetic properties of the calcined ore increased gradually. When the roasting time is prolonged more, the magnetic properties of the roasting ore decrease. When the roasting time is less than 30min, insufficient reduction occurs. But when the roasting time is too long, excessive reduction and waste of resources occur. Therefore, the most suitable time for roasting is 30min. Under this condition, the saturation magnetization of the calcined ore is 60.13emu·g^-1^.

### 3.2 Phase transformation analysis of magnetizing roasting

The magnetism of matter is closely related to the composition of matter and microstructure. In order to further study the phase changes of the mineral in the roasting process and the cause of magnetism changes, the calcined ore obtained under different conditions were investigated by XRD.

#### 3.2.1 Influence of temperature on phase transition

**Fig 11:**
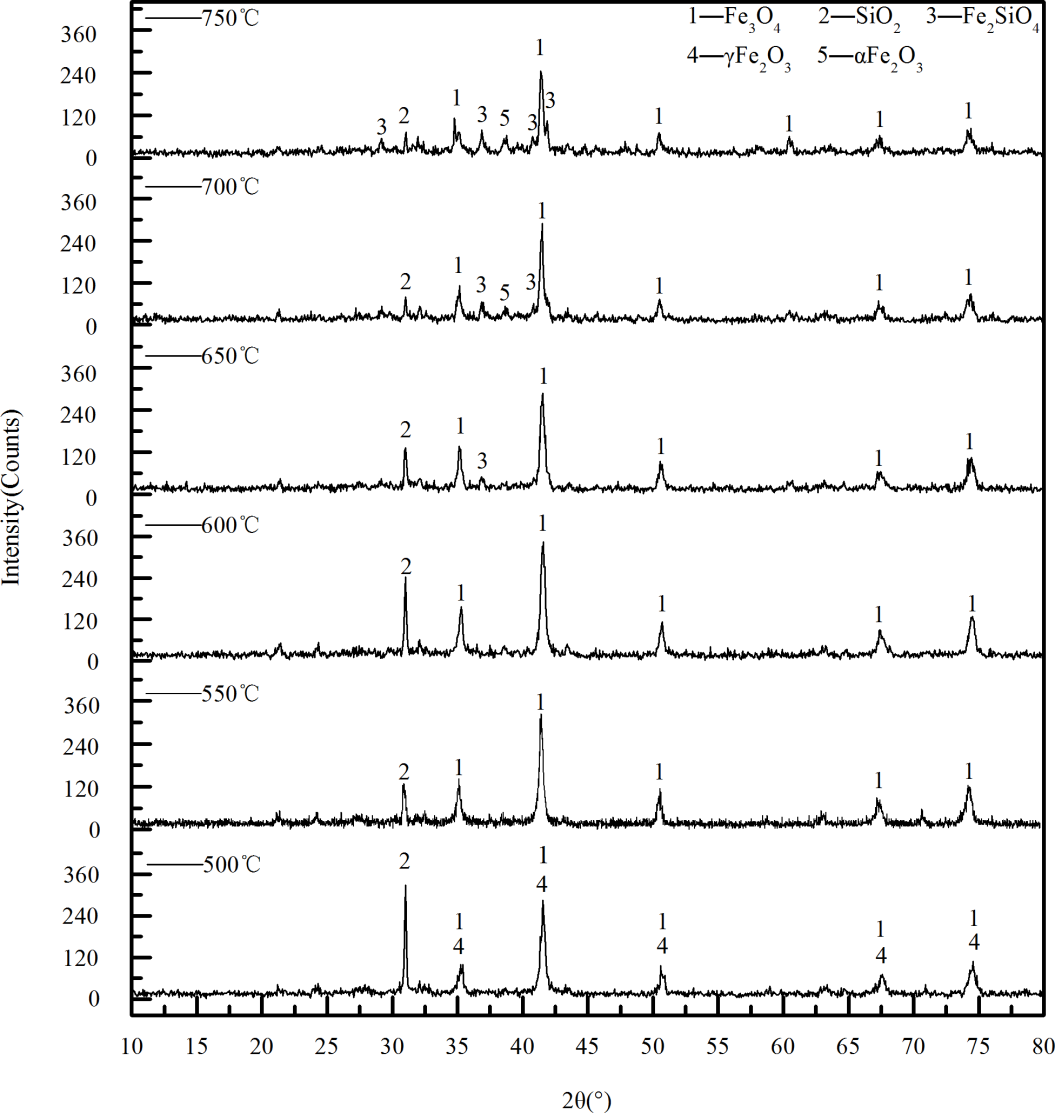
The XRD pattern of calcined ore obtained under different temperature.

Fig 11 shows the XRD pattern of calcined ore obtained at different temperatures, the other conditions are the biomass ratio of 25% and the calcination time of 45 min. Compared with the XRD curve of the raw ore, there are obvious Fe_3_O_4_ peaks. It can be inferred that there are a lot of Fe_3_O_4_ in the calcined ore. The Fe_3_O_4_ peak and γ-Fe_2_O_3_ peak exist at 500°C.The phenomenon has also occurred in the W.U. Yan et al[13] study on magnetic roasting of goethite results. It shows that the temperature is not enough to make all Fe_2_O_3_ be reduced into Fe_3_O_4_. When the temperature rose to 550°C, γ-Fe_2_O_3_ peaks disappeared, the intensity of Fe_3_O_4_ peak is strengthened, and the peak is sharper. This could explain the reason of hardly changes of the magnetism between 500~550°C. When the temperature continues to rise, in the XRD pattern of 650°C, there is a Fe_2_SiO_4_ peak at 2θ = 36.90°, and the intensities of the SiO_2_ peak at 2θ = 31.0° and the Fe_3_O_4_ peak at 2θ = 41.50° decreased. This indicated that the reaction temperature is too high, resulting in Fe_3_O_4_ reduction to ferrous oxide, which combined with SiO_2_ to form Fe_2_SiO_4_.So the magnetic component in the calcined ore reduced, resulting in weakening of the magnetic properties of the calcined ores. When the temperature rose to 700°C, the intensity of the SiO_2_ peak decreased, and there a α-Fe_2_O_3_ peak appeared at 2θ = 38.52°. When the temperature continued to rise to 750°C, the intensity of α-Fe_2_O_3_ peak was strengthened. This is because there is the reaction (4). This is consistent with the thermal decomposition of jarosite. In addition, from the XRD pattern of the calcined ore under 750°C, it can be seen that the Fe_2_SiO_4_ peak occurred at 2θ = 29.20°, 36.90°, 41.10°, 41.85° and 61.52°, and the intensity of the Fe_2_SiO_4_ peak got to be strengthened at 2θ = 36.90°. This indicates that with the increase of temperature, more and more magnetic material got to be converted into Fe_2_SiO_4_, which is consistent with the decrease of the saturation magnetization between 550~750°C in Fig 8.

#### 3.2.2 Effect of the content of reducing agent on phase transition

**Fig 12:**
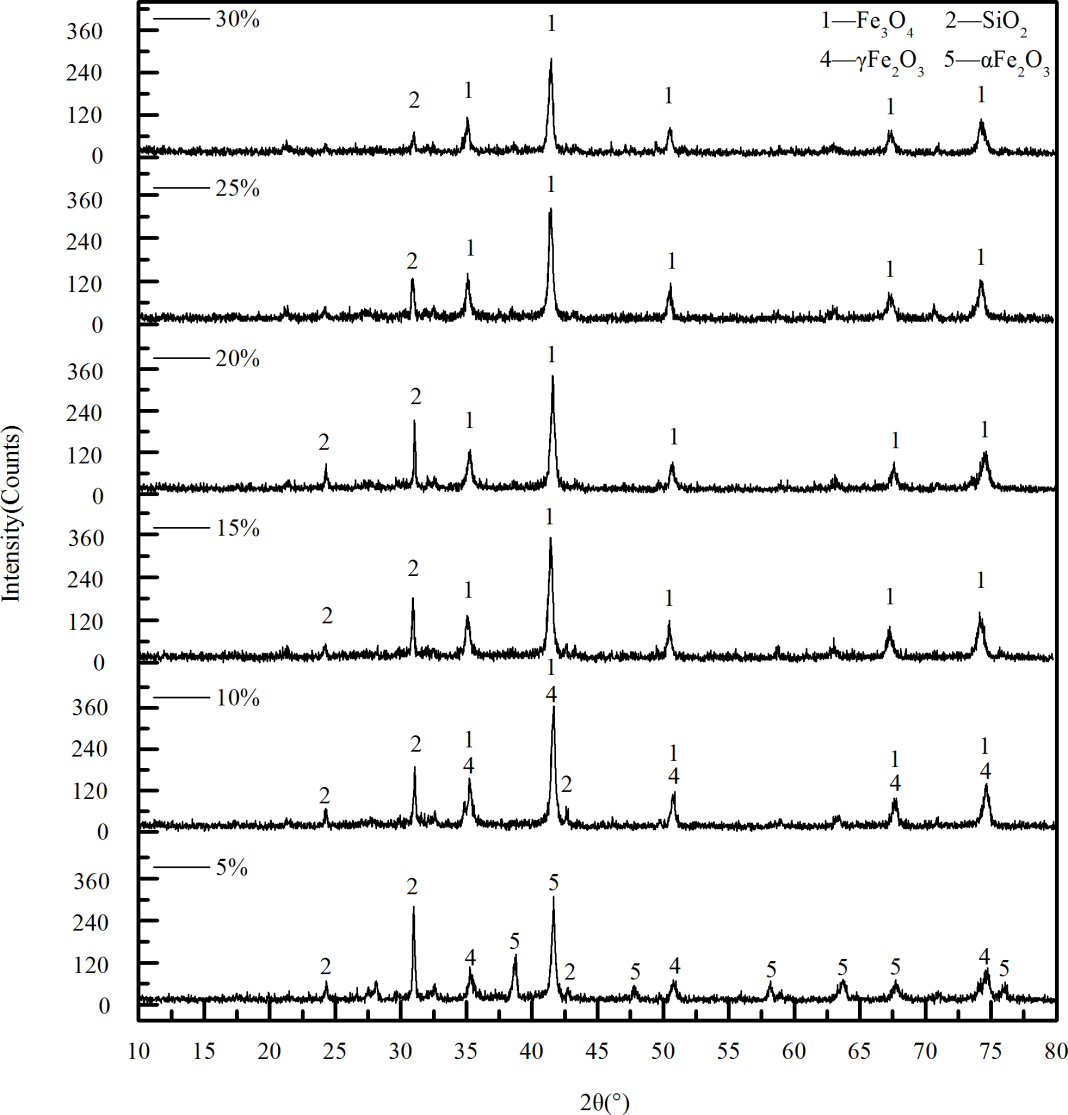
XRD patterns of calcined ore obtained under different content of biomass.

The influence of reducing agent content on the phase transformation of limonite at roasting temperature of 550°C and roasting time of 45min is shown in Fig 12. When the content of the biomass is 5%, there were obvious α-Fe_2_O_3_ peak at 2θ = 38.74° and 41.66°. This indicated that a part of α-Fe_2_O_3_ which could not be reduced still existed in the calcined ore. When the reducing agent content is 10%, the α-Fe_2_O_3_ peak at 2θ = 38.74°,2θ = 47.78° and 2θ = 76.01° disappeared, while the peaks at 2θ = 41.16° and 2θ = 74.60° got to be strengthened, and the peak was more sharp. This indicated α-Fe_2_O_3_ was further reduced. When the content of biomass continued to increase to 15%, no obvious change appeared in the position of the peaks, but the intensity of the Fe_3_O_4_ peak was enhanced and the peak shape is sharper, indicating the crystallization degree of material was better. This is consistent with a rapid increase in magnetic properties of the 5%~15% with the increase of biomass. As the biomass content continues to increase, the location of each peak is not obvious changes, but the intensity of the peak decreases.

#### 3.2.3 Effect of roasting time on phase transition

**Fig 13:**
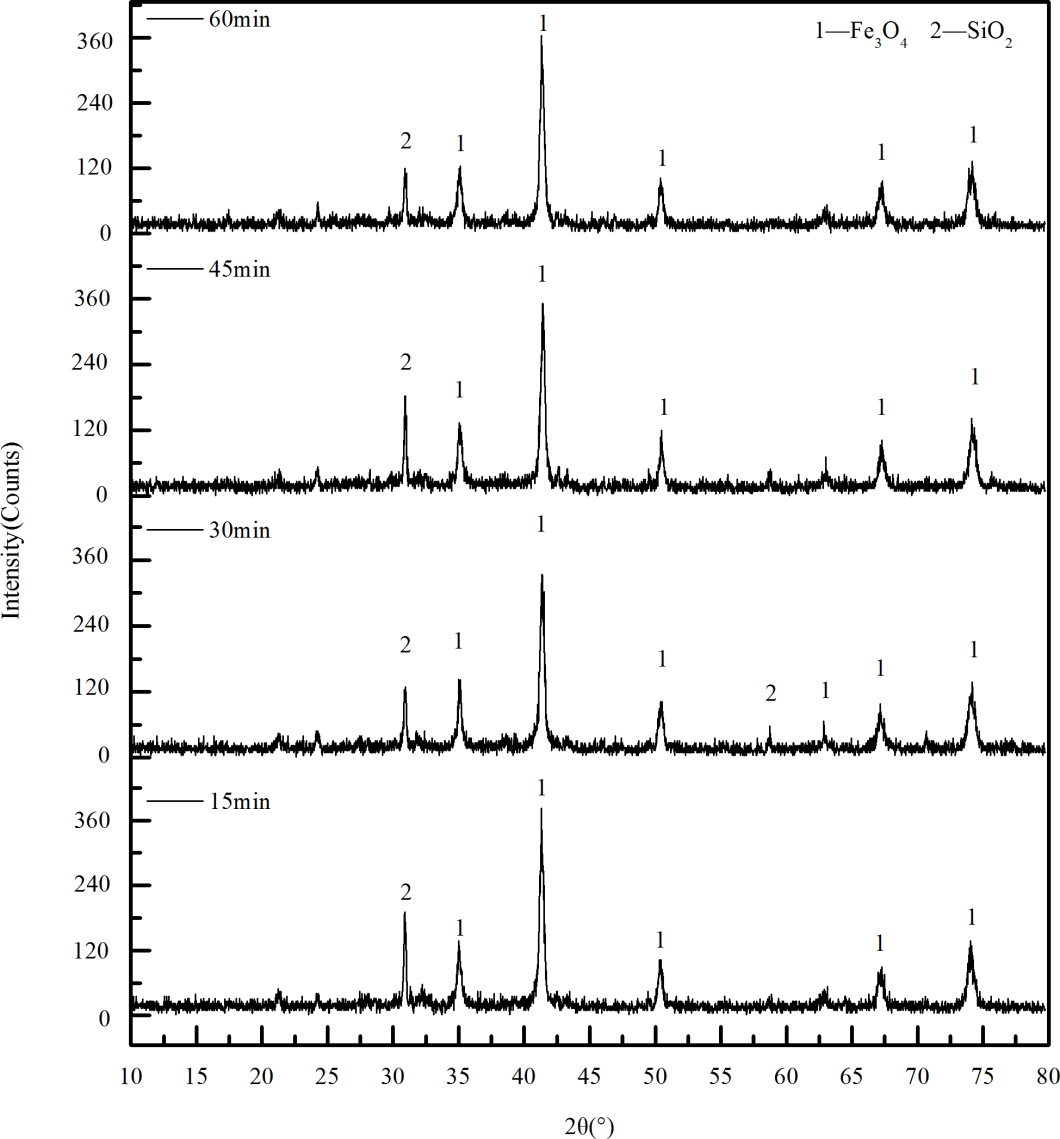
The XRD pattern of calcined ore obtained under different roasting time.

Fig 13 shows the XRD patterns of calcined ore at different roasting time, at a biomass ratio of 15% and a roasting temperature of 550°C. As can be seen from the diagram, the phase composition of calcined ore is not greatly changed with the roasting time between 15~60min. The main components of the calcined ore are Fe_3_O_4_ and SiO_2_. Therefore, the use of magnetic separation can effectively recover iron-bearing substances to obtain high-grade iron ore.

### 3.3 Analysis of morphology of calcined ore

**Fig 14:**
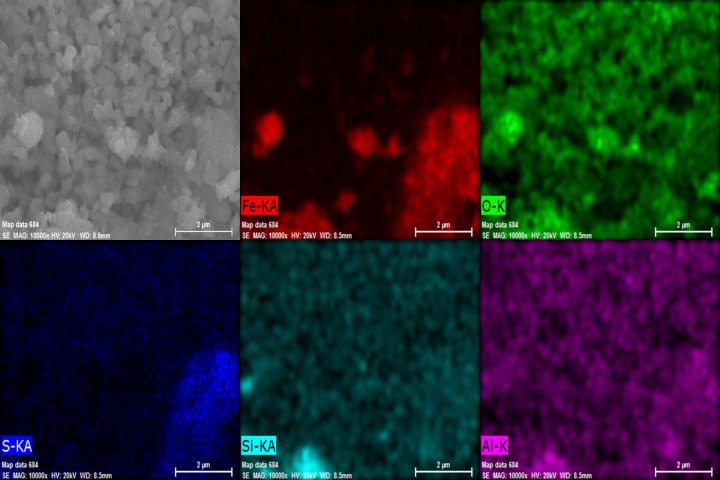
SEM image of calcined ore and distribution of elements in the calcined ore.

In order to observe the morphological difference of mineral particles between the raw ore and the calcined ore, the morphology of the calcined ore under the optimum condition was studied by SEM. Distribution of the main elements of the calcined ore were shown in Fig 14. As compared with the raw ore of Fig 2, the iron-bearing materials in the calcined ore becomes relatively concentrated and the clear boundaries between iron particles and slag particle are easily seen after roasting. The phenomenon indicated that there was the obvious growth of iron-bearing materials and the size of iron-bearing materials formed varies. However the other minerals containing silicon and aluminum becomes relatively uniform and smaller. In addition, a part of distribution areas of S coincides with the distribution area of Fe in the element distribution maps, it may be because there are some Fe_2_(SO_4_)_3_ in calcined ore.

## 4. Conclusion

Magnetic roasting of Guyang limonite by using biomass as reducing agent could promote the formation of ferromagnetic (Fe_3_O_4_). During roasting process, the iron-bearing materials in the limonite would first become α-Fe_2_O_3_ after dehydration at high temperature, and then α-Fe_2_O_3_ was reduced to Fe_3_O_4_ by biomass reduction. When the temperature is higher than 650°C, Fe_3_O_4_ will convert to Fe_2_SiO_4_, resulting in the reduction of magnetic materials and magnetism weakening of the calcined ore. When the roasting temperature is 550°C, the biomass ratio is 15% and the roasting time is 30min, the magnetic properties of calcined ore are the strongest, the saturation magnetization reaches 60.13emu·g^-1^, the recovery rate is 72%, and the iron grade is 58%.
